# OP27 Artificial Intelligence Use In Health Technology Assessment In Low- And Middle-Income Countries

**DOI:** 10.1017/S0266462324000904

**Published:** 2025-01-07

**Authors:** Maíra Catharina Ramos, Helena Eri Shimizu, Ivan Ricardo Zimmermann

## Abstract

**Introduction:**

Health technology assessment (HTA), by investigating clinical, economic, and social consequences of technologies in a country, enhances health system equity and sustainability. In low- and middle-income countries (LMICs), economic constraints and inadequate access to specialized human resources present challenges. Therefore, strategies to optimize resource allocation in the health sector are necessary.

**Methods:**

A literature review was carried out, with studies that directly identified barriers or facilitators for the use of artificial intelligence (AI) in HTA being considered eligible. The texts were analyzed from the perspective of LMIC. The searches were carried out on 8 August 2023 using the following databases: MEDLINE via PubMed, Web of Science, and Google Scholar. The selection was performed in two stages: (i) screening by title and abstract and (ii) evaluation of the eligibility criteria in full text.

**Results:**

After conducting the search, five studies were selected for narrative synthesis. Evidence of the potential benefits of using AI in HTA in low- and middle-income countries includes rationalization of resources; reduction of the burden on health systems and minimization of human workload; efficiency in data analysis, including clinical data; prediction of economic impact; and support for managerial decision-making. However, important challenges were also raised, such as the deficiency of local infrastructure; the training and education of professionals; the lack of ethical regulation; and the organizational and political considerations of these countries.

**Conclusions:**

There are few studies in the literature that provide scientific support on the use of AI in HTA decision-making in LMIC. The evidence points to increasing the efficiency and rationality of resources, enhancing the results arising from HTA. With this, it is expected to expand access to health technologies and enable more sustainable health systems.

